# Smoking Status Effect on Inflammatory Markers in a Randomized Trial of Current and Former Heavy Smokers

**DOI:** 10.1155/2015/439396

**Published:** 2015-08-23

**Authors:** Sami Aldaham, Janet A. Foote, H.-H. Sherry Chow, Iman A. Hakim

**Affiliations:** ^1^College of Medicine, Al-Imam Mohammad Ibn Saud Islamic University (IMSIU), Riyadh 11432, Saudi Arabia; ^2^Mel & Enid Zuckerman College of Public Health, University of Arizona, Tucson, AZ 85724, USA; ^3^Arizona Cancer Center, University of Arizona, Tucson, AZ 85724, USA

## Abstract

*Background*. The level of systemic inflammation as measured by circulating levels of C-reactive protein (CRP) and interleukin-6 (IL-6) is linked to an increased risk for cardiovascular diseases (CVD) and cancer. *Methods*. We recruited 154 current and former smokers between 40 and 80 years of age with 25 or more pack-years of smoking history to study the relationship between inflammatory markers (CRP and IL-6) and smoking status. *Results*. Our results show that male smokers had significantly higher levels of serum IL-6 compared to male former smokers. We did not find any gender specific differences for smoking and CRP levels but the IL-6 levels were slightly lower in females compared to males. Additionally, our results show that CRP is significantly associated with IL-6 regardless of smoking status. Modelling indicates that the significant predictors of CRP levels were biomarkers of the metabolic syndrome while the significant predictors of IL-6 levels were age and plasma triglycerides among former smokers and the numbers of smoked packs of cigarettes per year among smokers. *Conclusions*. In conclusion, our study showed that CRP levels were not associated with markers of smoking intensity. However, IL-6 levels were significantly associated with smoking especially among current smokers.

## 1. Introduction

Inflammation is associated with a number of chronic conditions, such as cardiovascular disease and cancer. Reducing inflammation may help prevent or treat these conditions. C-reactive protein (CRP) and interleukin-6 (IL-6) are well-studied proinflammatory cytokines [1, 2]. Inflammation is part of our immune reaction and leads to the release of C-reactive protein (CRP) into the bloodstream [3, 4] and IL-6 is a major factor driving the chronic elevation of CRP [5]. Elevated levels of IL-6 are predictive of future adverse health events in both healthy and clinical populations, even after controlling for both lifestyle and other clinical risk factors [6–9].

Smoking is the major risk factor for lung cancer, the most common cancer worldwide. Cigarette smoking has been associated with increases in CRP and previous investigations have demonstrated that increased CRP levels are a secondary effect of cigarette smoking and reflect tissue injury [10, 11]. The potential significance of IL-6 and CRP has been suggested in the growth and progression of many malignancies [12–14]. Many large-scale epidemiological studies among apparently healthy men and women have found CRP to be an independent and strong predictor of future cardiovascular risk [15–17].

There is great interest in studying the relationship between inflammatory markers and smoking in an attempt to provide explanations for smoking-mediated morbidity and mortality. The current study is unique with the inclusion and comparison of verified former smokers with a similar smoking history (pack-years and years of smoking) to the current smokers. The profile of inflammation markers is expected to differ following smoking cessation. Therefore, we are reporting here the associations of CRP and IL-6 with smoking status among community-dwelling men and women.

## 2. Methods

Current smokers (CS) and former smokers (FS) between 40 and 80 years of age with 25 pack-years or more of smoking history were recruited to a randomized, double-blind, and placebo controlled chemoprevention trial of green tea and black tea. The study protocol received approval by the University of Arizona Institutional Review Board. Once informed consent had been obtained, each participant completed a set of smoking, dietary, and health assessment questionnaires and was evaluated for health and respiratory history. A blood sample was collected for a comprehensive metabolic panel. Two additional fasting morning blood samples were collected from which plasma, serum, buffy coat, and erythrocytes were isolated and stored at −80°C prior to the sample analysis. Body mass index (BMI) for each participant was calculated as weight divided by height^2^ (kg/m^2^).

### 2.1. Human C-Reactive Protein (CRP) Immunoassay and Interleukin-6 (IL-6) Immunoassay in Serum

Aliquots were thawed immediately prior to the analysis. Each immunoassay was completed using a commercially available kit from Quantikine (R & D Systems, Minneapolis, MN, USA). Briefly, specified quantities of the specimen or standard are added to microplates with cells precoated with the CRP antibody or the IL-6 antibody, depending on the assay. The antibodies bind the CRP or IL-6 present in the samples and standards. Any unbound substances are washed away and a monoclonal antibody specific for CRP or a polyclonal antibody specific for IL-6 is added. Again, unbound substances are washed away, substrate solution is added, the plate is incubated, and amplifier solution is added. Color develops in proportion to the amount of CRP or IL-6 present in the well and after stopping the color development in the prescribed time, the wells are measured spectrophotometrically for CRP or IL-6 quantification. Both CRP and IL-6 analyses were completed in duplicate for 154 participants. All data entry and values were checked by additional laboratory personnel for quality control.

### 2.2. Statistical Analysis

Means and standard errors were calculated for descriptive statistics. Because of value skewness, CRP and IL-6 values were log-transformed. Pearson correlations were calculated to describe the unadjusted relationships between the smoking parameters, inflammatory markers, and other relevant variables. Multivariable linear regression models were created to identify independent predictors of log (CRP) and log (IL-6), using variables that had significant correlations (*p* < 0.10) with these inflammatory markers. A basic model was created for each inflammatory marker that included age, sex, BMI, caloric intake, triglycerides, high-density lipoprotein cholesterol, years of smoking, and cigarette pack-years (cigarettes smoked daily ÷ 20 cigarettes per pack × years of smoking).

## 3. Results

### 3.1. Population Characteristics

One hundred and fifty-four current and former smokers (83 women and 71 men) with a mean age of 60.3 ± 0.7 years (mean ± SE) were included in the study. Characteristics of the study population by gender and smoking status are presented in Table 1. There were no significant differences between males and females except in reported pack-years among current smokers. Male smokers reported significantly greater pack-years of smoking compared to females (*p* = 0.004). While the cumulative exposure smoking index of pack-years was not significantly different between current and former smokers among males or females, former smokers reported significantly fewer years of smoking compared to current smokers in both genders (*p* ≤ 0.001).

### 3.2. Inflammation Biomarkers

The differences in the levels of the biomarkers of inflammation and blood lipids between smokers and former smokers are presented in Table 2 for both men and women separately. Male current smokers had significantly higher levels of serum IL-6 compared to male former smokers. Women (smokers and former smokers) had significantly higher levels of HDL compared to men.

Given the fact that there were no significant differences by gender, the correlations among CRP, IL-6, smoking intensity parameters, and other variables are presented by smoking status in Table 3. Log (CRP) and log (IL-6) were significantly correlated regardless of smoking status (*p* < 0.001). Log (CRP) was not associated with any marker of smoking intensity. Among former smokers, log (CRP) was significantly associated with BMI (*r* = 0.280, *p* = 0.007) and low HDL levels (*r* = −0.273, *p* = 0.011). Among smokers, log (IL-6) was significantly associated with age (*r* = 0.466, *p* < 0.001), years of smoking (*r* = 0.242, *p* = 0.047), pack/years (*r* = 0.256, *p* = 0.033), plasma triglycerides (*r* = 0.268, *p* = 0.028), and low HDL levels (*r* = −0.029, *p* = 0.017). The log (CRP) was only associated with age (*r* = 0.270, *p* = 0.026).

### 3.3. Models of CRP and IL-6 by Smoking Status

The multivariate predictors of inflammatory markers by smoking status are presented in Table 4. The best multivariable model for log (CRP) among former smokers included triglycerides (*p* = 0.009) and the interaction between CRP and IL-6 (*p* < 0.001) and the best multivariable model for log (CRP) among current smokers included HDL (*p* = 0.010), BMI (*p* = 0.05), and the interaction between CRP and IL-6 (*p* < 0.001). The best multivariable model for log (IL-6) among former smokers included triglycerides (*p* = 0.002), age (*p* = 0.007), and the interaction between CRP and IL-6 (*p* < 0.001) and the best multivariable model for log (IL-6) among current smokers included pack/year (*p* = 0.005) and the interaction between CRP and IL-6 (*p* < 0.001).

## 4. Discussion

In the current study among participants with more than 30 pack-years of smoking exposure, we did not find any significant difference in CRP levels between smokers and former smokers. However, the serum levels of IL-6 were significantly higher among male smokers compared to former smokers (*p* = 0.04). Exposure to cigarette smoke increases oxidative stress which may lead to vascular inflammation. Because of the possible link between smoking and the initiation of inflammatory pathways, a large number of studies in which serum CRP concentrations have been measured in parallel to smoking status have been published in recent years [2–4, 10, 16]. However, some of these studies provided conflicting results [18]. The complexity of cytokine-mediated inflammation is described in a study showing that although smoking status was associated with a significant elevation in IL-6 levels, the increase in CRP levels observed in smokers was not statistically significant [19]. Another study indicated that mean CRP levels were significantly lower in never-smokers (*p* < 0.0001) compared to current smokers [20]. One study conducted in people of Japanese ethnicity did not find any significant relationship between serum CRP levels and the number of cigarettes smoked per day [4].

The MONICA study from Germany examined gender specific differences for smoking and CRP levels, finding that serum CRP concentrations were significantly higher in male regular smokers than male never-smokers but no significant differences were observed in women [21]. In our study we did not find any gender specific differences for smoking and CRP levels but the IL-6 levels were slightly lower in females compared to males. Also, women had significantly higher levels of HDL compared to men. In addition, our results showed that among smokers, CRP was associated with age while IL-6 was significantly associated with age, smoking variables as measured by years of smoking and number of pack-years, and dyslipidemia as measured by low HDL and high triglycerides levels.

There is strong evidence that IL-6 serum concentration increases with age [22, 23] and it is associated with an increased risk of cardiovascular disease (CVD) mortality, independent of CRP level [24]. Our data showed that the significant predictors of IL-6 levels among former smokers were age and plasma triglycerides. Among smokers, the smoking intensity as measured by the number of smoked packs of cigarettes across the number of years smoked was the main predictor of IL-6 high levels.

Smoking cessation lowers risk for lung and other cancer types along with reducing the risk of coronary heart disease, stroke, and peripheral vascular disease [25]. Inflammation is a key pathological component in these disease processes. Yet in this study among heavy smokers (mean of almost 43 packs per year for 33 years), CRP levels did not differ between former and current smokers. Additionally, former smoker IL-6 levels were significantly lower than the level among current smokers only among the males. These results suggest that CRP levels and IL-6 levels may not be the primary modulators of health improvements following cessation. Alternatively, intense smoking (high frequency over multiple decades) may have more longstanding effects on some types of inflammation. Our CRP results are consistent with results of Asthana et al. among smokers with a similar three-decade history of smoking but with half the pack-years [26].

Numerous studies have now confirmed that CRP levels are elevated in patients with the metabolic syndrome. CRP levels were shown to be strongly associated with BMI, low HDL, triglycerides, and levels of the proinflammatory cytokine, IL-6 [6, 27, 28]. Our results that showed that CRP levels were significantly influenced by HDL, triglycerides, and IL-6 levels support a potential relationship between CRP levels and the metabolic syndrome.

In conclusion, our data supports previous studies that showed that serum CRP is associated with biomarkers of the metabolic syndrome but not with smoking. However, serum IL-6 levels are mainly affected not only by the metabolic syndrome but also by age and smoking status.

## Figures and Tables

**Table 1:** Characteristics of the study population by gender and smoking status (*n* = 154).

	Males *n* = 71 mean ± SE	Females *n* = 83 mean ± SE	*p* value^*∗*^ (males versus females)
Age (years)	60.8 ± 1.0	59.9 ± 1.0	0.54
Former smokers	62.4 ± 1.5	60.9 ± 1.4	0.45
Current smokers	59.5 ± 1.3	59.2 ± 1.4	0.89
*p* value (former versus current)^*∗*^	0.14	0.40	
Pack-year	45.6 ± 2.6	40.7 ± 1.9	0.12
Former smokers	41.1 ± 3.4	44.6 ± 3.5	0.49
Current smokers	49.2 ± 3.7	37.7 ± 1.9	0.004
*p* value (former versus current)^*∗*^	0.12	0.07	
Years of smoking	34.4 ± 1.1	32.6 ± 1.2	0.28
Former smokers	29.5 ± 1.5	27.6 ± 1.6	0.39
Current smokers	38.4 ± 1.3	36.4 ± 1.5	0.33
*p* value (former versus current)^*∗*^	<0.001	0.001	

^*∗*^ *χ* ^2^ test.

**Table 2:** Biomarkers of inflammation and lipid profile by gender and smoking status (*n* = 154).

	Males *n* = 71 mean ± SE	Females *n* = 83 mean ± SE	*p* value (males versus females)
Serum C-reactive protein^*∗*^	7.4 ± 0.13	7.4 ± 0.11	0.66
Former smokers	7.2 ± 0.20	7.3 ± 0.16	0.58
Current smokers	7.6 ± 0.17	7.4 ± 0.16	0.27
*p* value (former versus current)	0.10	0.87	
Serum interleukin-6^*∗*^	0.67 ± 0.10	0.43 ± 0.10	0.09
Former smokers	0.44 ± 0.14	0.33 ± 0.13	0.57
Current smokers	0.86 ± 0.13	0.50 ± 0.14	0.08
*p* value (former versus current)	0.04	0.40	
Total cholesterol (mg/dL)	193.2 ± 5.1	206.2 ± 4.0	0.04
Former smokers	189.5 ± 6.7	204.6 ± 5.5	0.08
Current smokers	196.1 ± 7.6	207.3 ± 5.7	0.23
*p* value (former versus current)	0.52	0.74	
High density lipoproteins (mg/dL)	47.0 ± 1.5	61.5 ± 1.6	<0.001
Former smokers	47.1 ± 2.3	59.0 ± 2.7	0.002
Current smokers	47.0 ± 1.9	63.4 ± 1.9	<0.001
*p* value (former versus current)	0.99	0.17	
Low density lipoproteins (mg/dL)	114.0 ± 4.2	119.7 ± 3.6	0.30
Former smokers	111.8 ± 5.3	118.9 ± 4.6	0.32
Current smokers	116.8 ± 1.9	120.4 ± 5.3	0.67
*p* value (former versus current)	0.99	0.83	
Total triglycerides (mg/dL)	175.0 ± 18.5	139.5 ± 7.1	0.06
Former smokers	165.4 ± 18.6	142.5 ± 11.2	0.28
Current smokers	182.9 ± 30.3	137.3 ± 9.2	0.12
*p* value (former versus current)	0.64	0.71	

^*∗*^Log transformed data for C-reactive protein and interleukin-6.

**Table 3:** Pearson's correlation of CRP^*∗*^ and IL-6^*∗*^ with each parameter by smoking status (*n* = 154).

Variables	CRP		IL-6	
	*r*	*p*	*r*	*p*
Interleukin-6 (pg/mL)				
Former smoker	0.387	0.000	1.000	
Current smoker	0.589	0.000	1.000	
Age (years)				
Former smoker	0.161	0.140	0.198	0.067
Current smoker	0.270	0.026^‡^	0.446	0.000^‡^
Years of smoking				
Former smoker	0.081	0.461	0.202	0.062
Current smoker	0.017	0.889	0.242	0.047^‡^
Pack/year				
Former smoker	0.136	0.212	0.313	0.003^‡^
Current smoker	0.028	0.823	0.256	0.033^‡^
Caloric intake/day				
Former smoker	0.206	0.065	0.014	0.904
Current smoker	0.092	0.474	0.054	0.677
Total fat intake (g/day)				
Former smoker	0.173	0.126	0.007	0.951
Current smoker	0.090	0.488	0.124	0.338
Total cholesterol (mg/dL)				
Former smoker	0.024	0.825	0.024	0.829
Current smoker	0.134	0.275	0.086	0.484
HDL-C (mg/dL)				
Former smoker	−0.273	0.011^‡^	−0.207	0.056
Current smoker	−0.131	0.287	−0.290	0.017^‡^
LDL-C (mg/dL)				
Former smoker	0.028	0.799	0.072	0.517
Current smoker	0.182	0.141	0.061	0.623
Total triglycerides (mg/dL)				
Former smoker	0.168	0.123	0.134	0.220
Current smoker	0.196	0.110	0.268	0.028^‡^
BMI (kg/m^2^)				
Former smoker	0.287	0.007^‡^	0.129	0.236
Current smoker	0.203	0.100	0.130	0.299

CRP: C-reactive protein; HDL-C = high density lipoproteins; LDL-C: low density lipoproteins; BMI: body mass index.

^*∗*^Log transformed data for CRP (ng/mL) and IL-6 (pg/mL).

^‡^Significant correlation.

**Table 4:** Stepwise regression analysis for CRP and IL-6 by smoking status.

Subjects	Explanatory variable	*β*	*p*	Adjusted *R* ^2^
CRP (mg/L)				
Former smokers	Total triglycerides	0.496	0.009	0.455
	CRP **∗** IL-6	<0.001	0.000	
Smokers	HDL-C (mg/dL)	−0.017	0.010	0.369
	BMI (kg/m^2^)	0.028	0.052	
	CRP **∗** IL-6	<0.001	0.000	
IL-6 (pg/mL)				
Former smokers	Total triglycerides	0.442	0.002	0.547
	Age	0.027	0.007	
	CRP **∗** IL-6	<0.001	0.000	
Smokers	Pack/year	0.012	0.005	0.436
	CRP **∗** IL-6	<0.001	0.000	

CRP: C-reactive protein; IL-6: interleukin 6; HDL-C: high density lipoprotein; BMI: body mass index, and CRP **∗** IL-6: interaction between CRP and IL-6.

Variables included in the model are gender, age, BMI, total triglycerides, HDL-C, years of smoking, pack/year, caloric intake/day, and interaction between CRP and IL-6.

## Highlights

- We conducted a cross-sectional study on a sample of 154 current and former smokers.
- We investigated the relationship between inflammatory biomarkers and smoking status.
- CRP levels were significantly associated with IL-6 levels regardless of smoking status.
- The significant predictors of CRP levels were biomarkers of the metabolic syndrome.
- IL-6 levels were significantly associated with smoking especially among current smokers.
